# Development and Characterization of Electrodes for Surface-Specific Attenuated Total Reflection Two-Dimensional Infrared Spectroelectrochemistry

**DOI:** 10.1021/acs.jpcc.3c05445

**Published:** 2023-11-28

**Authors:** Melissa Bodine, Vepa Rozyyev, Jeffrey W. Elam, Andrei Tokmakoff, Nicholas H. C. Lewis

**Affiliations:** † Department of Chemistry, James Franck Institute, and Institute for Biophysical Dynamics, The University of Chicago, Chicago, Illinois 60637, United States; ‡ Pritzker School of Molecular Engineering, The University of Chicago, Chicago, Illinois 60637, United States; § Applied Materials Division, Argonne National Laboratory, Lemont, Illinois 60439, United States

## Abstract

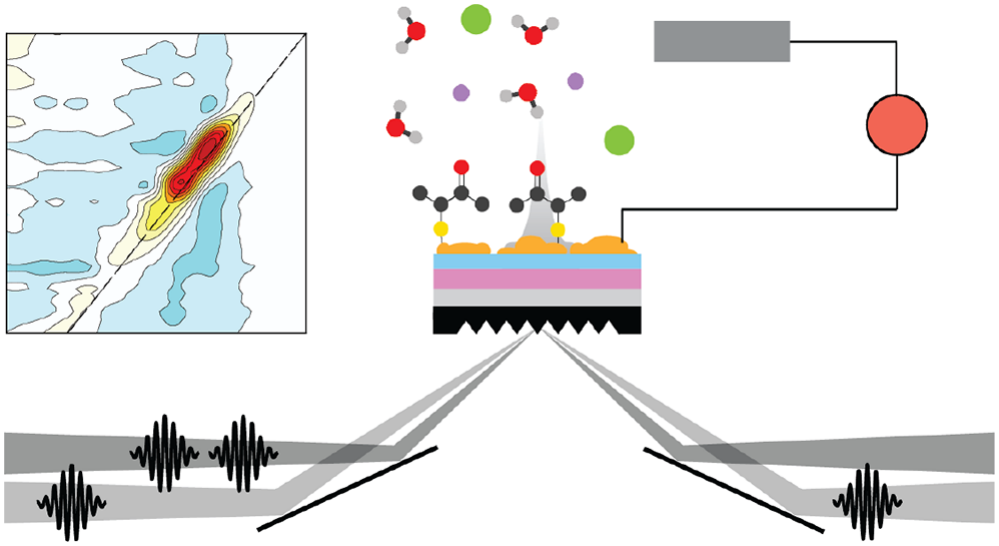

Electrochemical interfaces still have remaining mysteries surrounding the interfacial region of the electrical double layer, despite being prevalent throughout the energy and water remediation industries. The electrical double layer is where many important dynamic processes such as catalysis and electron transfer occur. The goal of this work is to study the electrical double layer with two-dimensional infrared (2D IR) spectroscopy to experimentally access the details of the structural dynamics of this complex environment. However, there are several experimental challenges to applying 2D IR spectroscopy to this application, such as assuring the surface specificity of the spectrum, optimizing the signal strength while minimizing spectral distortions from dispersion and Fano line shapes, and selecting electrode materials that are both sufficiently IR compatible and conductive. Here we will discuss various considerations when designing 2D IR experiments of electrode interfaces utilizing several substrates and experimental configurations and demonstrate a robust method for 2D IR experiments of electrode interfaces under applied potential that combines nonconducting Si ATR wafers with conductive ITO and thin nanostructured films of plasmonically active Au functionalized with 3-mercapto-2-butanone (MCB). We show that layered electrodes on thin Si ATR wafers with MCB are sensitive to applied potential and that the distortions in the linear and 2D IR spectra are heavily dependent on the morphology of the Au surface.

## Introduction

The properties of the electric double layer’s structure and dynamics at solid–liquid interfaces are still not fully understood, despite being vitally important to a wide class of problems. These problems include developing efficient materials for water remediation, where controlling specific adsorbate–adsorbent interactions is important for selective filtration and recycling of water-soluble finite resources, and range to understanding the impact of fluctuations in the electric double layer (EDL) on important electrochemical processes such as catalysis and electron transfer.^1−3^ The examples mentioned above all have similar questions remaining, related to how the water in the EDL dynamically restructures to accommodate solutes being adsorbed and desorbed, surface reactions, and interfacial charge transfer. Investigating these problems remains difficult because of the complex interplay between the solution, solutes, and electrochemical interface that imparts a large electric field on the system and complicates interpretation of experiments.^4^

The advantage of using vibrational spectroscopy to study solid–liquid interfaces is the detailed information it can provide related to molecular structure, and by combining vibrational spectroscopy with electrochemical techniques, it is possible to specifically isolate small changes in the species that are impacted by the surface potential.^5^ There is a long history of the development of surface-enhanced IR (SEIRAS), Raman (SERS), and sum frequency generation (SFG) methods for vibrational spectroelectrochemical measurements,^6−14^ with the scientific focus ranging from problems in catalysis to voltage-sensitive biomolecules and fundamental properties of solutions at an electrified interface.^15−22^ With the addition of ultrafast techniques, such as 2D IR, it is possible to obtain additional insights into the dynamics of the system on the time scales of molecular vibrations.^23,24^ Of particular interest to us is the change in the hydrogen-bonding structure and dynamics at electrode–electrolyte interfaces and the dynamical role of water in electrochemical processes. Additionally, specific ion effects influence the EDL properties, and understanding the origin of these influences can help to inform the design of more efficient electrode–electrolyte systems for important applications such as CO_2_ reduction.^25^

Due to the intrinsically low oscillator strength for molecular vibrations, there are several intrinsic challenges when using IR spectroscopy to gain interface-specific information. One of the major approaches for overcoming these challenges is through the use of tethered or adsorbed molecules at the interface, which provides surface specificity, and the inclusion of a plasmonic metal, usually Au or Ag, to provide local electric field enhancement, which enhances the magnitude of the signal.^9,26,26,27^ The highest enhancement factors for IR absorption are found in small nanogaps between the nanostructures where the electric field is magnified greatly.^28−31^ One of the difficulties of plasmonic enhancement is the possibility of phase-twisted Fano line shapes that can complicate the interpretation of the surface-enhanced spectra in both linear and 2D IR spectra. Additionally, experimentally investigating solid–liquid interfaces with 2D IR remains a difficult task due to complications that arise when using ultrafast pulses in conjunction with traditional substrates for SEIRAS. This is especially a concern for 2D IR experiments of electrochemical interfaces because typical bulk conductors and semiconductors can give rise to large transient reflection background signals from excited free carriers.

The Kretschmann configuration, whereby the working electrode is deposited as a thin film on the surface of the optical element for attenuated total internal reflection (ATR), is preferred when integrating FTIR and electrochemical studies because of the advantages in the design of the electrochemical cell.^8,23^ In addition to simplifying the inclusion of the electrodes necessary for electrochemical measurements, one of the main advantages of this experimental geometry when working with small signals from monolayers of vibrational probes is to reduce the solvent background absorption by restricting it to the micrometer-scale penetration depth of the evanescent wave rather than the full path length through a transmission cell. For these reasons, we focus our discussion primarily on substrate materials that can be used as ATR elements, although we also provide comparison with transmission measurements. Typically, when selecting IR ATR materials, the criteria are broad transparency in the IR, a high index of refraction, and a large effective path length or penetration depth. When working with femtosecond pulses, the common ATR materials like Si and Ge can have large and long-lived transient responses when in the beam focus due to the multiphoton excitation of free carriers across the relative low bandgaps of these materials. There is also a trade-off between a material’s reflective losses and the index of refraction—while a higher index of refraction lends greater flexibility to the incident angle and compatible solvents by decreasing the critical angle for total internal reflection—it also leads to greater reflective losses, which may be detrimental for signal intensities in nonlinear spectroscopic measurements. Conversely, common materials used for 2D IR experiments such as CaF_2_ have a low index of refraction, limiting their use as ATR materials when working with electrolyte solutions that have indices of refraction near to or greater than that of pure water at 1.33 due to the large critical angle required for total internal reflection. For electrochemical experiments where the conditions can degrade the electrodes from the current cycling as well as concentrated electrolyte solutions which can alter pH, the robustness and chemical compatibility of the ATR material are also a consideration. Some of the more robust materials are diamond, which is cost inhibiting and has interfering phonon resonances across the IR region, and Si. CaF_2_ is easily damaged and likely requires regular repolishing between experiments, and materials like ZnSe are soft and can potentially leach ions into the solution. It is also valuable to minimize the material in the beam path to minimize temporal dispersion, which further complicates the experimental configuration.

To address these concerns, we have tested multiple potential ATR substrates—ZrO_2_, CaF_2_, and Si—for viability by sputtering thin films of Au onto the substrates that are used both for plasmonic enhancement and as the reactive layer to functionalize the surface with 3-mercapto-2-butanone (MCB), which acts as a local vibrational probe of the interface. The linear and 2D IR spectra are measured to compare the relative performance of the different substrates. We furthermore measure the linear-IR spectra as a function of Au film thickness to determine the relationship between the film thickness intensity and the signal of the MCB carbonyl stretch mode. Previous studies have shown that vibrational probes such as CO and nitriles are sensitive to the interfacial electric field and demonstrated that their utility in studying the properties of electrochemical interfaces.^14,32−35^ We choose a carbonyl as our vibrational probe because the frequency can be correlated to the local electrostatic field^36−38^ and does not suffer from the same complications as nitriles in hydrogen-bonding solvents where there are contributions from both the π and σ hydrogen-bonding interactions.^39,40^

For the final electrode design for our experiments, we have developed a layered electrode that can be used in the Kretschmann ATR geometry and takes into account all of the challenges of performing 2D IR spectroelectrochemistry at the electrode–electrolyte interface. We choose Si as a substrate because of its versatility as a universal substrate for surface studies, which makes it a good base for fabricating many types of electrodes. ITO is used as a conductive layer, with a thin layer of Al_2_O_3_ acting as an adhesion layer for nanostructured Au which is functionalized with MCB. We evaluate the electrochemical performance of the electrodes, demonstrate the changes in the linear-IR and 2D IR spectra as a function of applied potential, and determine the potential of zero charge to provide a meaningful reference potential for measured IR spectra.

## Materials and Methods

### Materials

We evaluated three IR-compatible materials to serve as substrates for our experiments. Cubic zirconia (ZrO_2_) ATR prisms (10 mm × 10 mm prisms with an 80° apex angle) were obtained from Supply Chain Optics. Undoped Si wafers micromachined for use as ATR IR substrates (Universal ATR Crystal, 11 mm × 9 mm × 0.5 mm) were obtained from IRUBIS. Throughout this study, we refer to these as Si wafers. The 25 mm ϕ × 1 mm thick CaF_2_ windows were obtained from Crystran. Prior to deposition of Au or electrode materials, the substrates were cleaned by rinsing in DI water and then submerging in methanol for 15 min and dried under a stream of nitrogen.

3-Mercapto-2-butanone (MCB), NaCl, and solvents were purchased from Sigma-Aldrich and used as received. D_2_O (99.9%) was purchased from Cambridge Isotope Laboratories. Sulfuric acid, hydrochloric acid (36.5–38%), and nitric acid were purchased from Fisher Scientific. Atomic layer deposition (ALD) reactants, including cyclopentadienylindium(I) (InCp), tetrakis(dimethylamino)tin(IV) (TDMASn), and trimethylaluminum (TMA), were purchased from Strem and used as received.

### Surface Fabrication

In addition to measurements using the fully layered electrode that we developed, we performed a series of tests using only Au deposited directly onto the substrates. Gold was deposited with a Cressington 208HR sputter coater equipped with a quartz crystal microbalance to control the average film thickness. A 99.99% Au target (Ted Pella) was used with a 40 mA current for an average deposition rate of 3.7 nm/min. Following sputter coating, samples were submerged overnight in an ethanol solution of 50 mM MCB to functionalize the Au, subsequently rinsed gently in DI water and ethanol, and dried under a stream of nitrogen.

For the full layered electrode fabrication, indium tin oxide (ITO) and Al_2_O_3_ thin films were grown by atomic layer deposition (ALD) in a custom viscous flow reactor, the details of which have been described previously.^41^ ALD films were grown at 0.7 Torr under constant vacuum with an ultrahigh-purity nitrogen gas flow of 225 sccm mass flow rate and at a constant reactor temperature of 250 °C. For ITO deposition, InCp and TDMASn were reduced with ozone with pulse/purge times of InCp:ozone 3 s/5 s:5 s/10 s and TDMASn:ozone 2 s/10 s:2 s/10 s.^42,43^ During the reaction, InCp and TDMASn precursors were held in stainless steel bubblers at 45 °C to achieve sufficient vapor pressure for ALD reactions. For every 19 cycles of InCp:ozone there was one cycle of TDMASn:ozone, to obtain 3.3% Sn doping, confirmed by XPS. A total of 130 ALD cycles of ITO (In + Sn) were performed to deposit 20 nm of film, confirmed by ellipsometry measurements (J. A. Woolam Co., M2000V Vase). Al_2_O_3_ films were grown directly on top of the ITO layer in the ALD reactor using TMA and H_2_O as precursors with pulse/purge times of 1 s/10 s for both reagents. After ALD of ITO and Al_2_O_3_, the surfaces were cleaned as described above, and Au was sputtered on top of the Al_2_O_3_ layer and functionalized with MCB.

To remove the electrode materials from Si ATR wafers for reuse, the Au was wiped off, and then the oxide layers were removed by first submerging in 2% sulfuric acid on a 100 °C hot plate for 5 min and subsequently in aqua regia on a 60 °C hot plate for 15 min.

### Surface Characterization

Atomic force microscopy (AFM) images were obtained with a Bruker Multimode 5 AFM with BudgetSensor Tap300-G probes (Ted Pella). The images were processed with Gwyddion v2.61.^44^ Conductivity was measured with a Jandel 4-point probe.

XPS measurements of the Au 4f peaks at 84 and 88 eV were performed by using a Thermo Fisher K-Alpha+ spectrometer. The data were analyzed using Avantage software (Thermo Fisher), and the spectra were referenced to the adventitious C 1s peak at 284.8 eV. An average of five scans was presented for each reported spectrum. The X-ray source was a microfocused monochromatic Al Kα (1487 eV) beam with a spot size of 400 μm. For the survey scans, 200.0 eV with a step size of 1.000 eV of pass energy was utilized. When performing the high-resolution XPS measurements, 50.0 eV with a step size of 0.100 eV of pass energy was used.

### Electrochemistry

For electrochemical measurements, the ITO/Al_2_O_3_/Au/thiol monolayer samples on top of the Si ATR wafers were used as the working electrode in a Jackfish J2 spectroelectrochemical cell with a Ag/AgCl (3 M KCl) reference electrode and a Pt wire counter electrode. The Si ATR wafers are designed to be compatible with the Jackfish cell and are assembled as per the manual. A potentiostat (Gamry, Interface 1010E) was used to control the electrochemical potential of the sample.

Cyclic volammetry (CV) measurements were scanned between −300 and 500 mV, starting at −300 mV, with a scan rate of 1 V/s, and were repeated 10 times sequentially. FTIR spectroelectrochemical measurements were performed by taking the background at 0 mV vs Ag/AgCl and stepping the potential sequentially between 500 and −500 mV in 50 mV steps, and 2DIR spectroelectrochemical measurements were performed by measuring spectra at 300, 0, and −300 mV. For CV and spectroelectrochemical measurements, the supporting electrolyte was 100 mM KCl in D_2_O that had been purged with N_2_.

For potentiostatic electrochemical impedance spectroscopy (EIS) measurements, the applied AC voltage frequency *f* was scanned from 0.2 to 300000 Hz with 10 points taken per decade. The AC amplitude was set to 14.1 mV, and the default number of cycles per frequency step was used. The DC offset was scanned from 0.5 to −1 V in steps of 10 mV. The supporting electrolyte for the EIS measurements was 10 mM KCl in H_2_O that had been purged with N_2_.

### IR and 2D IR Spectroscopy

Linear absorption spectra were measured with a Bruker Tensor 70 Fourier transform IR spectrometer with a variable angle attachment for the ATR measurements (Pike, VeeMax III), set to a 50° angle of incidence.

For CaF_2_ substrates, all spectra were measured in transmission mode using two CaF_2_ windows with a 6 μm spacer to reduce the solvent background from D_2_O while keeping the windows from incident contact that could damage the Au thin film. Only the first CaF_2_ window in the beam path was coated to reduce reflection losses and interference from the back-reflection off of a second Au film. For CaF_2_ FTIR measurements, the background spectra were collected of air with the cell removed. ZrO_2_ prism and Si wafer FTIR spectra were measured in ATR mode by using a clean substrate as the background. For FTIR measurements with an applied potential, the background was taken at 0 mV vs Ag/AgCl. For Si ATR wafers, the micromachined grooves were oriented parallel to the beam path for the FTIR according to the manufacturer guidelines. For 2D IR measurements the micromachined grooves were oriented perpendicular to the beam path so that the beams were incident on only one face of the Si ATR wafer to reduce scatter.

Ultrafast 2D IR measurements were taken in the pump–probe geometry with a custom-built spectrometer described in detail elsewhere^45^ using mid-IR pulses centered at 1700 cm^–1^ with a full width at half-maximum bandwidth of 200 cm^–1^, 15 μJ/pulse, and 100 fs pulse duration. Briefly, the probe pulse is split from the pump using the front face reflection of an uncoated wedged CaF_2_ window. The waiting time τ_2_ between the pump and the probe is controlled by a delay stage (Aerotech) in the probe beam path. The pump beam is sent through a pulse shaper (Phasetech, Quickshape) that generates a phase-locked pulse pair with controllable delay τ_1_ and is used to compensate for dispersion.

The probe and pump beams were focused into the sample with 90° off-axis parabolic mirrors. For transmission measurements, the sample was placed directly in the beam focus of a 4 in. focal length mirror (150 μm spot size). For ATR measurements, we used a 6 in. focal length mirror (200 μm spot size at normal incidence) with the addition of two gold mirrors to create a 50° angle of incidence onto the ATR prisms. The pump intensity was attenuated (100–200 nJ per pump pulse) to prevent damage of the gold film and to mitigate the nonresonant response from the ATR element. The change in probe intensity induced by the pump is measured using a spectrograph (Horiba, Triax 190) equipped with a 64-element HgCdTe detector (IR Associates) to obtain the detection frequency axis.

For the transmission 2D IR measurements, the pump and probe were set to the *ssss* polarization. To minimize pump scatter contamination of the signal, all 2D IR ATR measurements shown were measured in the cross-polarized *ppss* configuration with the pump *p*-polarized and the probe *s*-polarized with respect to the interface. The polarization of the pump and probe beams was set by  waveplates (Alphalas) and wire grid polarizers (Thorlabs). The polarization of the detected signal is set to *s* with a wire-grid analyzer placed immediately before the spectrograph.

## Results and Discussion

### Evaluation of IR Substrates

We tested three potential ATR substrates: ZrO_2_, CaF_2_, and Si. ZrO_2_ has the advantage of a high index of refraction and in comparison to Si has a much larger bandgap, which reduces the probability of exciting free carriers in the substrate through multiphoton excitation. This is advantageous because the excitation of free carriers leads to large background signals. ATR prisms made from ZrO_2_ are also durable and can be reused extensively. However, ZrO_2_ is an uncommon optical material, necessitating custom manufacture of ATR elements. The absorption edge of ZrO_2_ also rises steeply below 1600 cm^–1^, which can lead to challenging signal isolation for carbonyl vibrational probes and large dispersion in this region of the spectrum, which must be carefully compensated with germanium.^46^ CaF_2_ avoids the problem of large dispersion in this frequency range but has a low index of refraction which is problematic for ATR measurements, particularly with aqueous solutions. The CaF_2_ spectra measured in this study are therefore in transmission mode, although we note that for certain solvents with lower indexes of refraction such as acetonitrile, or systems studied under air, CaF_2_ may be a good choice as an ATR substrate. The last ATR substrate we used was a commercial IRUBIS Si ATR wafer that is compatible with the Jackfish J2 spectroelectrochemical cell and the ATR FTIR attachment. The main concern for Si is the possible excitation of free carriers and potentially large reflective losses due to the high index of refraction, but with thin micromachined Si ATR wafers, the reflective losses and dispersion are minimal. With the addition of a plasmonic layer, we are also able to obtain surface sensitivity with pump intensities as low as 100–200 nJ per pump pulse (100 fs pulse duration), which is below the threshold for the multiphoton excitation of free carriers in Si.

**Figure 1:**
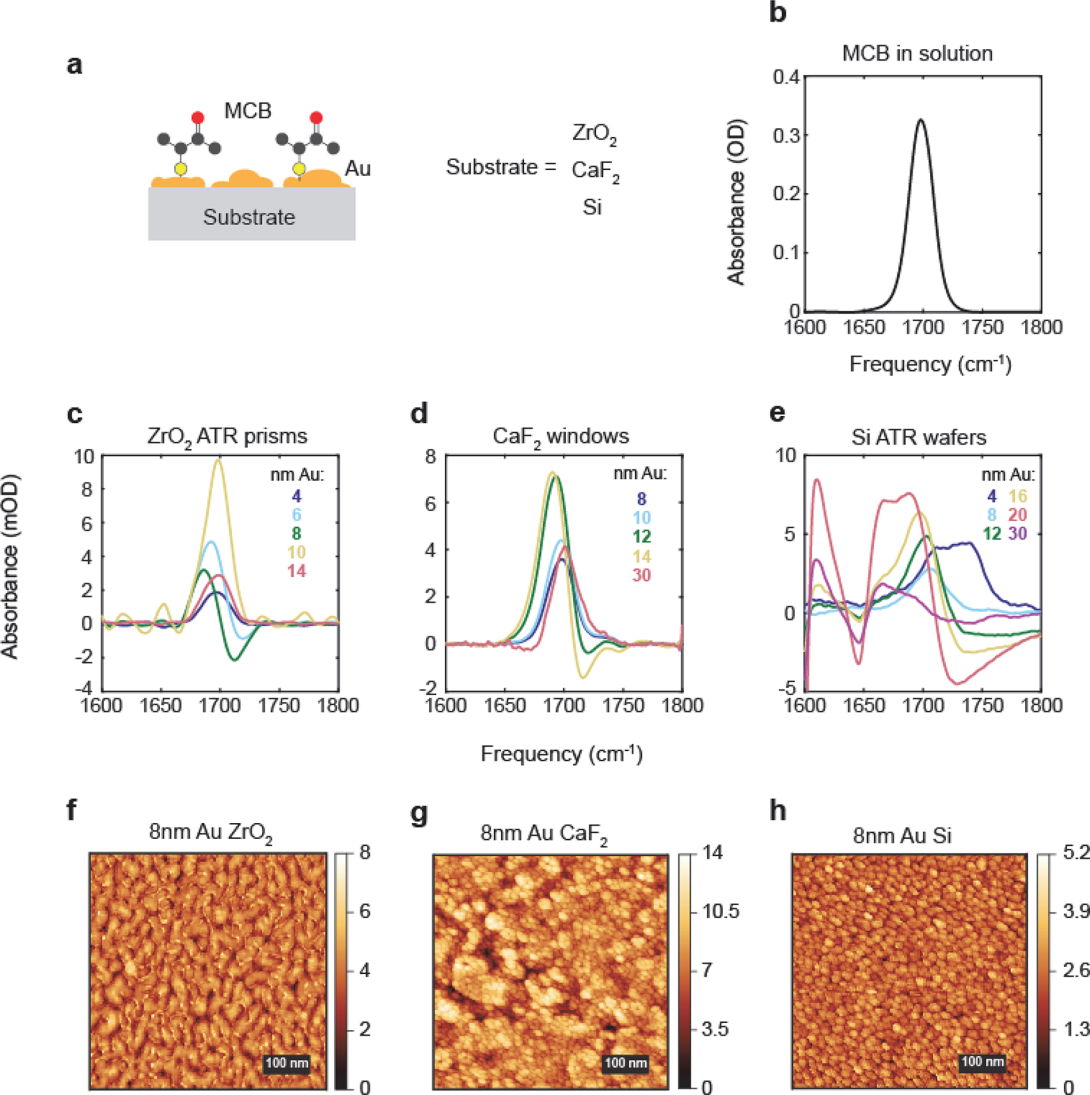
(a) Illustration of nanometer gold surfaces functionalized with MCB. (b) Bulk spectrum of MCB in D_2_O solution. (c–e) Background subtracted FTIR spectra of MCB on different sputtered thickness of Au (gold film thickness values (nm) are indicated in the inset) on ZrO_2_ ATR prisms, CaF_2_ windows, and IRUBIS Si ATR wafers with air (no solvent) above the sample. The FTIR with CaF_2_ substrate is measured in transmission geometry, while all other spectra are ATR mode. (f–h) AFM images of 8 nm Au sputtered on ZrO_2_, CaF_2_, and Si, respectively.

To achieve surface enhancement, we sputtered Au thin films onto the substrates. A cartoon illustrating the sample morphology is shown in Figure 1a, and a comparison spectrum of MCB in a solution of D_2_O is shown in Figure 1b. Sputtered surfaces have the advantage of facile fabrication as compared to more highly enhancing plasmonic nanoarrays^28−31^ as well as an increased surface roughness in comparison to other deposition methods.^47^ To optimize the signal from the molecular monolayer, we sputtered Au at several thickness ranging from 4 to 30 nm average thickness on each of the substrates and then functionalized the Au with MCB. We also investigated annealing and resputtering the Au films^48^ but found that there was no difference in the enhancement. To determine the magnitude of the absorbance by the vibrational probe with respect to Au film thickness, we measured the FTIR spectra of the MCB carbonyl stretch for each substrate and film thickness, shown in Figure 1c–e. The FTIR spectra were fit with a smooth polynomial baseline that excluded the carbonyl peak, and then the fit was subtracted to isolate the molecular response. For the Si FTIR spectra shown in Figure 1e, the overlapping peaks around 1600–1650 cm^–1^, which we assign to water adsorbed in the electrode material,^49,50^ complicate the fitting and thus are not included in background subtraction, and the background is fit excluding a wider range from 1500 to 1850 cm^–1^. Examples of the fitting and background subtraction for each substrate are shown in Figure S1.

For the MCB monolayers, the carbonyl resonance frequency is not greatly different from that of the MCB in aqueous solution, but the MCB spectra have distinct variations by substrate. As expected from previous studies,^27,51−53^ there are obvious Fano distortions to the line shape of the carbonyl peak and changes in intensity that vary as a function of Au thickness. We note that for sputtering, where the thickness of the film is the average thickness and the microscopic structure is highly inhomogeneous, there can be variations between samples prepared even when using the same method. This leads to changes in the enhancement and line shape between samples even when using the same preparation and film thickness. The Fano distortion appears as a red-shift in the maximum frequency and the appearance of a negative absorption dip on the blue side of the spectrum. For the Si substrates, as the Fano distortion increases, the peak is shifted closer to the overlapping peaks from adsorbed water, but the same negative absorption on the blue side and increasing/red-shifting peak absorbance is observed. The relationship between the line shape and Au thickness is dependent on multiple factors, but most importantly the coupling between the broad plasmon and the narrow vibrational mode.^54^

**Figure 2:**
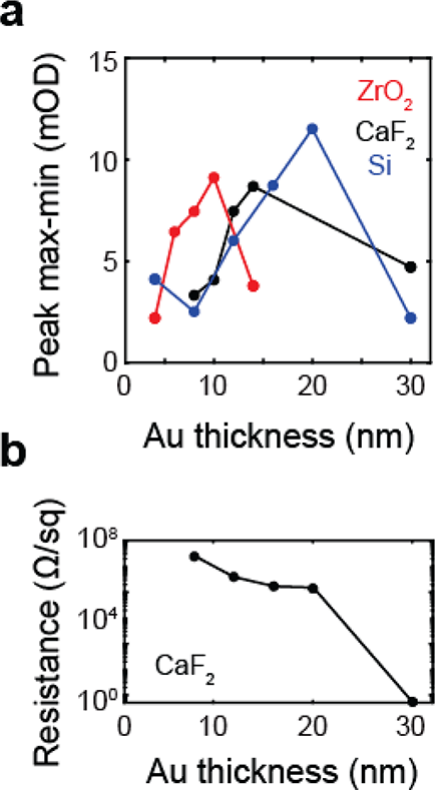
(a) Difference between maximum and minimum peak absorbance for MCB as a function of sputtered Au film thickness for three substrates: ZrO_2_, CaF_2_, and Si ATR wafers. (b) Sheet resistance as a function of sputtered Au film thickness on CaF_2_ windows.

We quantify the enhancement of the carbonyl peak by taking the difference between the maximum and minimum peak absorbance of MCB. We see in Figure 2a that the enhancement initially grows with Au thickness but reaches a maximum value between 10 and 20 nm before decreasing at higher Au coverage. The optimal Au thickness for maximizing the carbonyl peak enhancement is dependent on the substrate, with the ZrO_2_ showing a maximum response at 10 nm Au, 16 nm for the CaF_2_, and 20 nm for the Si. The decrease in carbonyl enhancement at high Au coverage is correlated to the sharp drop in resistance of the Au films, shown in Figure 2b, which become substantially more conductive with the increasing average thickness of the Au as the nanoislands percolate to form a connected surface.

The variation in the optimal Au thickness between the different substrates is likely due to the different percolation thresholds for Au for the various substrates, which results in differences in the morphology as a function of the average thickness. This is illustrated in Figure 1f–h, where we show AFM images of the Au films with 8 nm average thickness on each substrate. On ZrO_2_, the percolation threshold for the Au occurs at lower average thicknesses than the other two substrates and at 8 nm already shows merging of the nanoislands. In contrast, 8 nm Au on CaF_2_ and Si shows a distinct pattern of spherical nanoislands. The two competing factors that lead to the greatest enhancement are the overlap of the plasmon resonance into the IR region, which increases with the size of the particles and is maximized for bulk Au, and the presence and density of nanogaps where the field is greatly enhanced. When the percolation threshold is reached, the plasmon resonance overlap into the IR is greater than that of nanostructured Au films, but the presence of nanogaps is greatly reduced, resulting in a reduction in the signal from the monolayer.^27,55^ Because the enhancement of the monolayer signal is dependent on the coupling of the vibration to the plasmon, which is also the origin of changes to the carbonyl line shape, there will always be a trade-off between signal enhancement and carbonyl line shape changes. In this study, we ultimately chose to use 12 nm thick films of sputtered Au for our electrochemical experiments because it provides significant enhancement without the extreme distortions seen in the 20 nm thick Au films, but we recognize that this may not always be the best choice and expect that our results will help future researchers make informed decisions when designing interfacial IR experiments that rely on signal enhancement.

**Figure 3:**
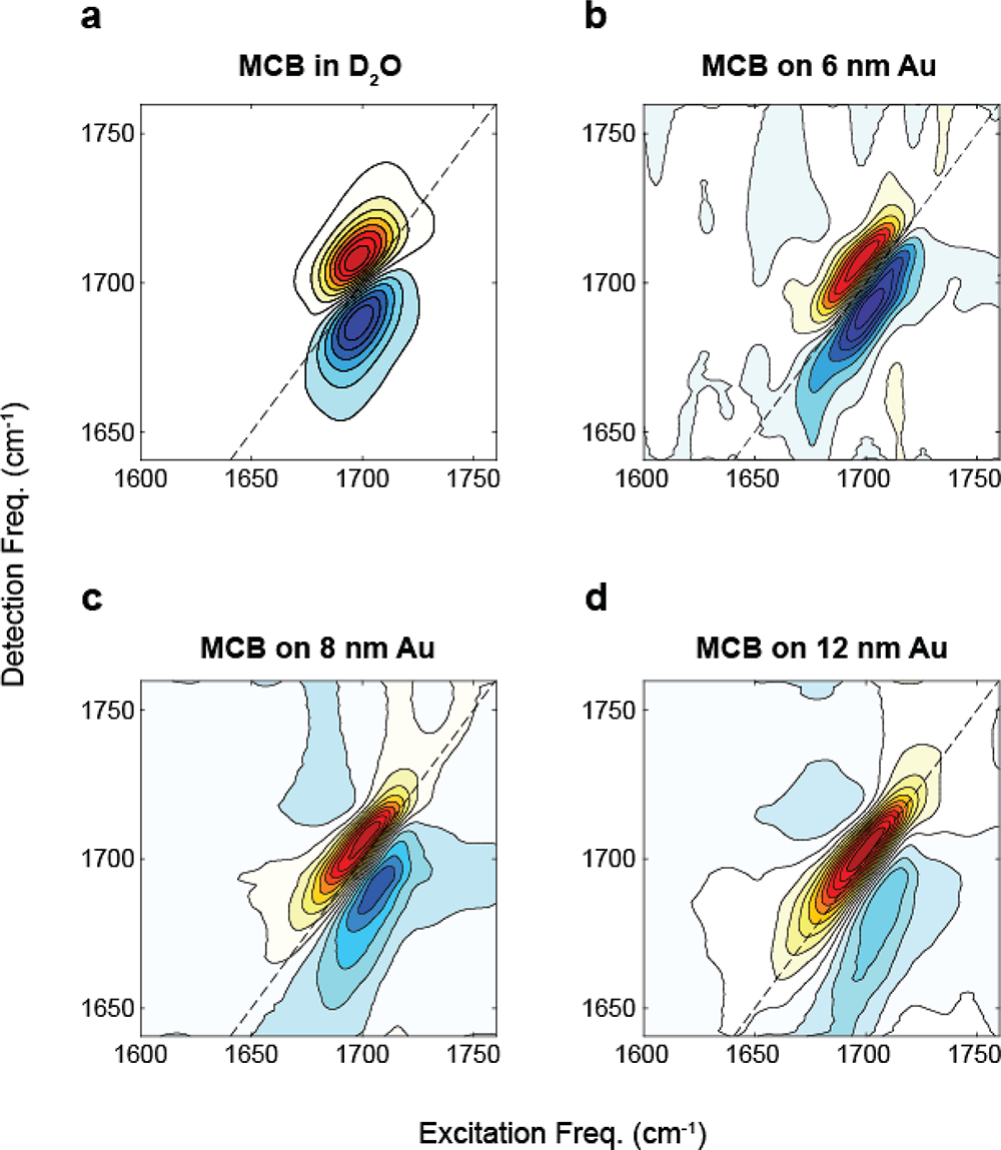
2D IR spectra of carbonyl stretching vibration of MCB acquired at *t*_2_ = 300 fs. (a) Bulk solution spectrum of 250 mM MCB in D_2_O. (b–d) Spectra of MCB functionalized on Au layers of various thickness on CaF_2_ at the interface with D_2_O: (b) 6, (c) 8, (d) 12 nm. Each spectrum is individually normalized.

We can further demonstrate the impact of the Au thickness by observing the effect on the 2D IR line shape as well as the changes on the surface as compared to the spectrum in aqueous solution, shown in Figure 3. In panel a, we show the spectrum of MCB in D_2_O, where we see the usual form of a 2D IR spectrum with the 0 → 1 bleach transition appears as the positive (yellow-red) band and the 1 → 2 induced absorption appears as the negative (blue) band, shifted to lower frequency along the detection axis by the vibrational anharmonicity. These bands are elongated along the diagonal of the spectrum due to the transient heterogeneity in solution at early waiting time. In Figure 3b–d we show the spectra for MCB functionalized on Au surfaces of increasing average thickness, which illustrate several differences in line shape relative to the solution spectrum. Compared to the solution spectrum, we see an increase in the diagonal line width with increasing Au coverage and a decrease in the antidiagonal homogeneous line width. One explanation is that this would indicate that the carbonyl groups of the MCB on the surface do not experience the same fluctuations as in solution, together with a larger degree of inhomogeneity due to local surface environments. For the 6 nm Au layer the spectrum is minimally phase-twisted showing equal intensity positive and negative peaks, whereas increasing Au coverage leads to a decrease of the negative intensity below the diagonal and growth of a negative feature above the diagonal. This is in tandem with changes in the signal intensity, with the 6 nm layer providing the least enhancement and the 12 nm layer the greatest, comparable to the effect on the FTIR spectrum. A more detailed investigation into the impact of plasmon–vibration coupling on vibrational spectra is beyond the scope of this paper but clearly needs to be explicitly addressed to fully interpret experimental results.

**Figure 4:**
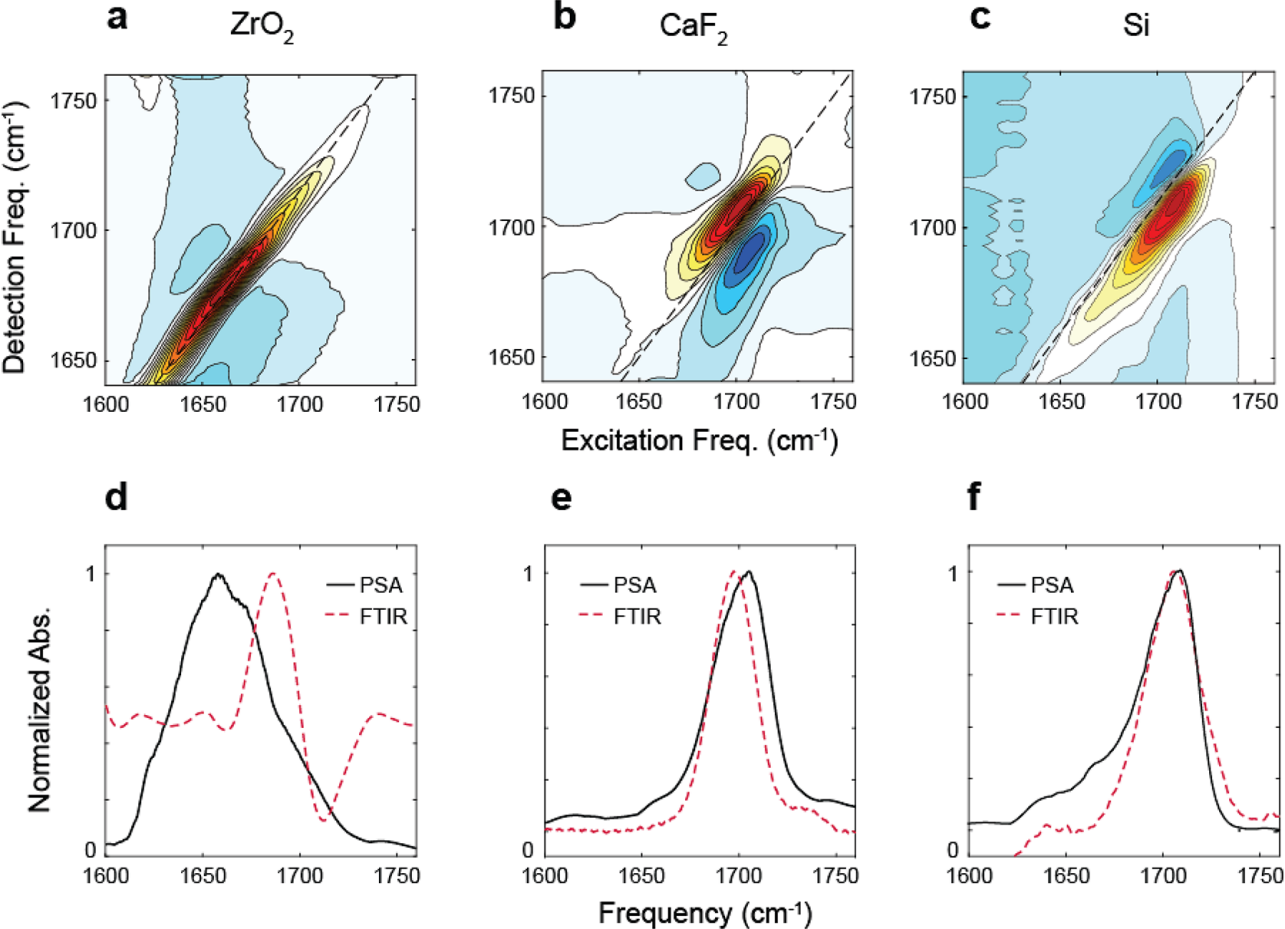
2D IR spectra of MCB-functionalized Au films on different substrates are shown in panels a–c for ZrO_2_, CaF_2_, and Si, respectively. The Au film thickness is 8 nm on ZrO_2_, 10 nm on CaF_2_, and 12 nm on Si. The lower panels d–f show the corresponding pump slice amplitudes (PSA) overlaid with the FTIR spectra for the corresponding system.

We also show the 2D IR spectra for MCB on each substrate in Figure 4a–c with Au film thicknesses of 8 nm for ZrO_2_, 10 nm for CaF_2_, and 12 nm for Si to illustrate the impact of the substrate. The different thicknesses of Au films for each substrate reflect the film thickness that maximized the enhancement of the spectra. Similar to the FTIR measurements, the differences in morphology of the Au film on each substrate influence the degree of Fano phase twist in the 2D IR spectra, although here the variation is further emphasized. The effect of the Au film on the 2D IR line shape is greatest for the example spectrum on Si in Figure 4c, where the ground state bleach/stimulated emission and excited state absorption peaks appear to be almost completely inverted. For ZrO_2_ (Figure 4b) and CaF_2_ (Figure 4c), the 2D IR spectra show a lesser amount of phase twisting, resulting in a negative–positive–negative pattern along the detection frequency axis rather than the conventional positive–negative pattern seen in solution spectra. This demonstrates that both the average Au thickness and the properties of the substrate are important for determining the appearance of the final spectrum. Comparing the FTIR of all three substrates, we observe that the center frequencies and line widths are similar, except for the ZrO_2_ FTIR which shows a large phase twist at the same average Au film thickness that can be attributed to different morphology of Au on ZrO_2_.

We can compare for each substrate the FTIR and the 2D IR by overlapping the pump slice amplitude^56^ (PSA) and FTIR as shown in Figure 4d–f. We see that while the PSA spectra for CaF_2_ and Si are similar to the FTIR, ZrO_2_ shows an extremely broadened diagonal line width and a large red-shift in the peak frequency. This is in contrast to the FTIR spectrum of the ZrO_2_ system, which is not dramatically dissimilar from the other substrates. We have previously used ZrO_2_ successfully for 2D IR ATR measurements of bulk solutions,^45^ so these unusual results on ZrO_2_ seem to be related in some way to the surface preparation. While the origin of the distortion of the 2D IR spectrum on ZrO_2_ is not entirely clear, it precludes the use of this substrate for 2D IR measurements of surface bound species. While CaF_2_ substrates show comparable results with the Si substrates, Si has the major advantage of being a universal substrate for many surface studies, and therefore there are a large range of functionalization and characterization techniques that have been developed for Si substrates. In this study we focus on developing one specific method that integrates ITO electrodes grown on Si and 2D IR and expect the principles used in this study to design the 2D IR compatible electrodes can be applied to the 2D IR studies of other Si-based interfacial systems that may require alternative electrode design. Therefore, we focus on the Si substrates for further spectroelectrochemical application.

### Electrode Fabrication on Si ATR Wafers

A conductive substrate is required for electrochemical experiments. Because the MCB signal also decreases significantly on conductive Au films, we use a layered approach to decouple the plasmonic enhancement from the conductivity of the electrode. Specifically, we incorporate a conductive layer of indium tin oxide (ITO) beneath thin plasmonic Au. This approach has previously been demonstrated for FTIR applications^57−59^ and more recently for 2D IR.^32,34^ We choose to use ALD to fabricate the ITO layer due to the atomic level control that it provides via self-limiting growth and its ability to grow high-quality, low-resistivity thin films.^42^ By separating the plasmonic material from the bulk conductor of the electrode, we are able to separately optimize each layer. However, this flexibility in the design comes at the cost of the additional complexity in the fabrication and additional considerations such as the quality of adhesion between the layers.

**Figure 5:**
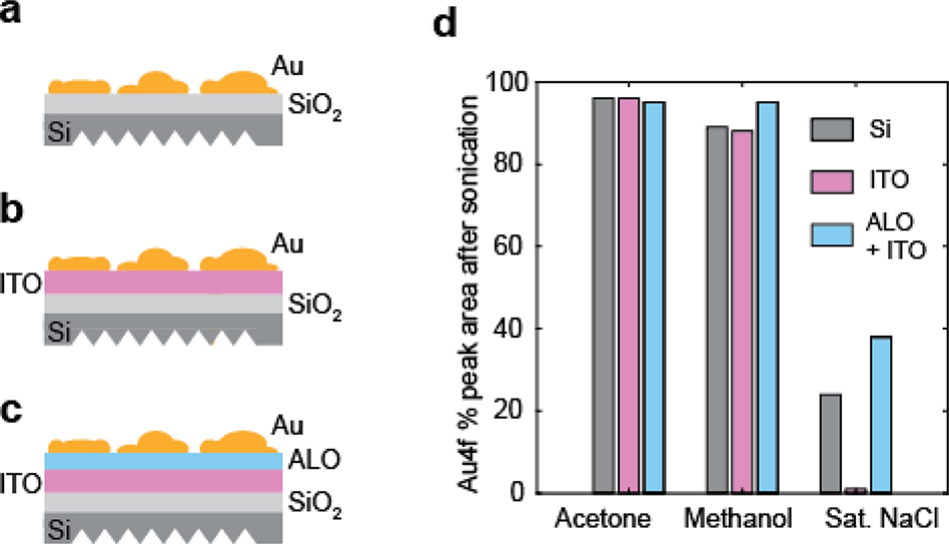
Adhesion of 14 nm sputtered Au on different fabricated substrates as quantified by XPS. Results for adhesion test shown are shown for (a) 14 nm of sputtered Au on bare Si, (b) ALD grown ITO on Si, and (c) ALD grown Al_2_O_3_ on ITO/Si. (d) XPS peak area normalized to the unsonicated case for three solvents: acetone, methanol, and saturated aqueous NaCl solution.

To improve the adhesion of the Au layer to the ITO, we incorporate a thin (1–2 nm) Al_2_O_3_ layer on top of the ITO, also grown through ALD, which covalently binds to the ITO and serves to increase the adhesion of the Au layer as compared to bare ITO.^60,61^ A cartoon illustration of the layered electrode construction is shown in Figure 5a–c. We tested the adhesion strength of Au to ITO vs Al_2_O_3_ and the bare Si substrate by sonicating a sample with a 14 nm layer of Au for 25 min in methanol, acetone, or a saturated aqueous NaCl solution. We then determined the subsequent loss of Au coverage with XPS, using the area of the Au 4f peak before and after sonication (Figure S2). This test was designed to simulate conditions harsher than spectroelectrochemical experiments, which are performed without sonication in a static 0.10 M KCl solution and should represent a worst case scenario for the loss of Au under normal operation. The results of this test are shown in Figure 5d. For organic solvents that are commonly used to clean surfaces or as the medium for the functionalization of Au with thiols, there is very little loss of Au after sonication. For the concentrated NaCl solution, the Au is completely removed after sonication when it was sputtered directly onto the ITO layer, as compared to 24% remaining on the bare Si and 38% with the thin layer of Al_2_O_3_. The AFM images showing the morphology of the different surfaces (ITO, Al_2_O_3_, and Si) as well as the images of the Au-coated surfaces before sonication are shown in Figure S3. Although we cannot determine if the Au film is being dissolved in solution or if the mechanical perturbation from sonication is leading to removal of the film, this test provides us with confidence that the inclusion of ITO and Al_2_O_3_ layers between the Si substrate and the plasmonic Au layer will result in a sufficiently robust electrode for our purposes. We also verified that the insulating Al_2_O_3_ layer was sufficiently thin to maintain a low resistivity. When the resistivity of the full electrode (layered ITO and 12 nm Au) was tested with and without the Al_2_O_3_ layer, the increase in sheet resistance was minimal (from 16 to 58 ohm/sq). Common alternative adhesion layers for Au on oxides include thin films of Ti or Cr, which should be investigated but may cause problems by reducing IR transmission through the electrode.

### IR and 2D IR Spectroelectrochemistry

**Figure 6:**
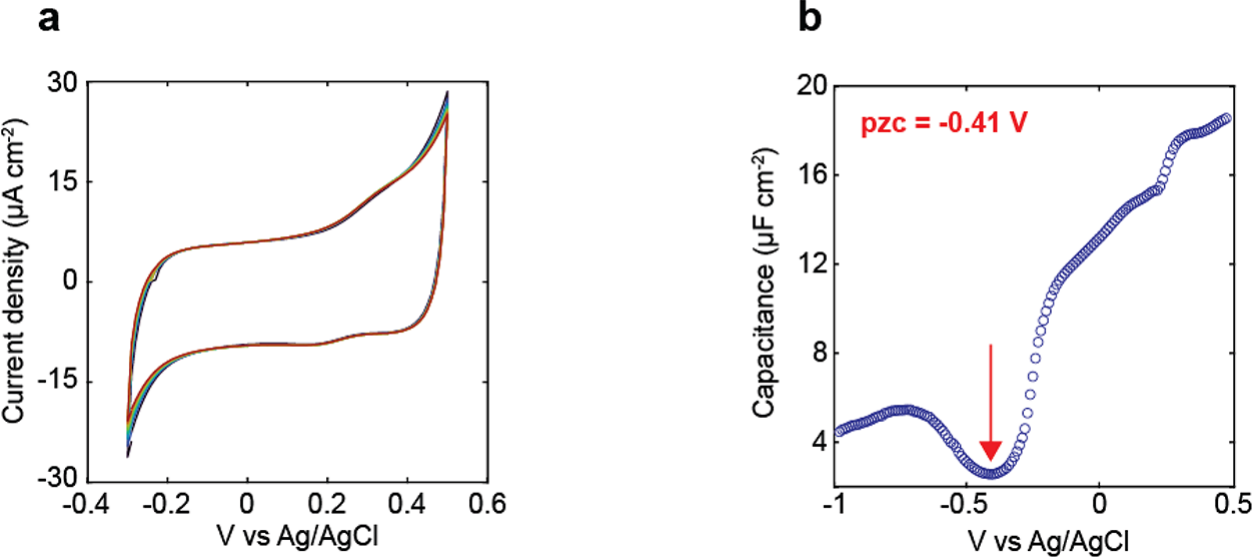
(a) Cyclic voltammogram of the layered electrode (12 nm Au/1.2 nm Al_2_O_3_/20 nm ITO on Si ATR wafers) functionalized with MCB which is shown to be stable over 10 cycles. (b) Capacitance determined from EIS measurements as a function of potential, where we assign the minimum in capacitance at −0.41 V vs Ag/AgCl to coincide with *E*_pzc_.

In Figure 6 we characterize the electrochemical performance of our MCB-functionalized electrodes before measuring the FTIR spectra as a function of the electrochemical potential. All experiments and spectra measured with an applied voltage are performed with a layered electrode fabricated on an Si ATR wafer with 20 nm ITO, 1.2 nm Al_2_O_3_, 12 nm Au, and functionalized with MCB. The CV, shown in Figure 6a, demonstrates a stability window from −300 to 500 mV, where minimal electron transfer processes are taking place, and a stable response over 10 cycles, demonstrating the electrochemical stability of the electrode and electrolyte over this potential window. The dominant response in the CV in this window comes from the charging and discharging of the EDL. We attribute the small redox wave centered at 0.25 V to chloride adsorption and desorption as they are not present when KClO_4_ is used as the electrolyte (Figure S5).

To choose a physically meaningful reference potential, we used electrochemical impedance spectroscopy (EIS) to determine the potential of zero charge (*E*_pzc_), the potential at which the electrode has on average no net surface free charge.^62,63^ To determine *E*_pzc_ from EIS measurements, the modulus of the impedance |*Z*(*E*,*f*)| was modeled and fit to a simplified Randles equivalent circuit, as shown in eq 1:
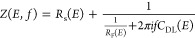
1Here, *E* represents the DC potential and *f* is the AC frequency of the potential, *R*_s_ is the uncompensated resistance of the solution, *R*_F_ is the Faradaic resistance, and *C*_DL_ is the double layer capacitance of the working electrode. *E*_pzc_ is then identified as the potential at which *C*_DL_ is minimized. An example fit for the impedance is shown in Figure S4. As shown in Figure 6b, *E*_pzc_ is observed at −410 mV. *E*_pzc_ serves as a meaningful reference due to the influence that the surface charge has on the interfacial properties of the electrode, particularly the state of the EDL. It has been associated with a change in the water orientation at the interface that has been probed with both IR and VSFG spectroscopy.^21,64^

**Figure 7:**
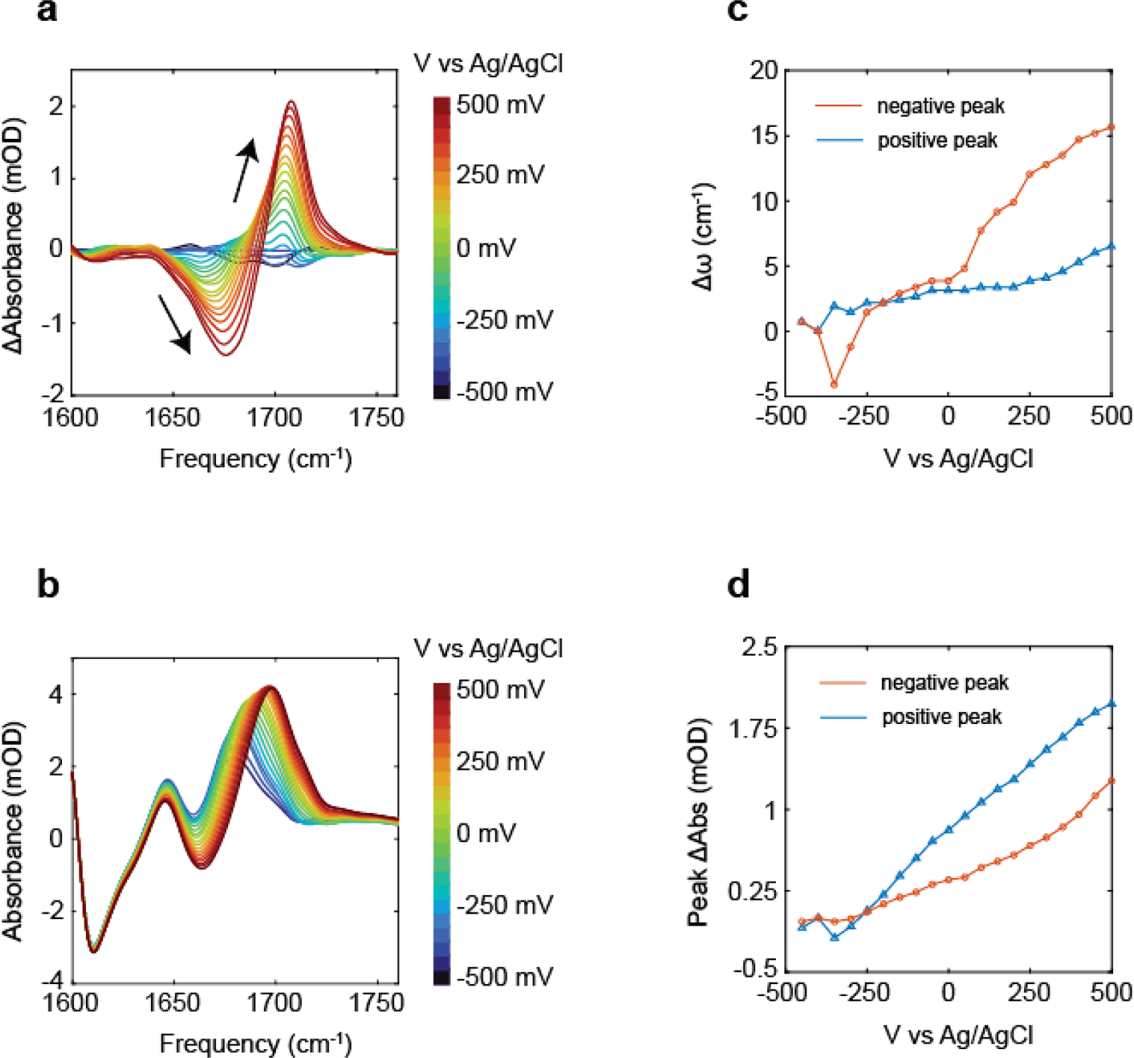
(a) FTIR difference spectra of MCB on 12 nm Au sputtered on Al_2_O_3_/ITO/Si substrates with respect to −400 mV vs Ag/AgCl reference electrode and 100 mM KCl supporting electrolyte in D_2_O. Difference spectra are additionally background subtracted to remove baseline shifts due to potential-dependent changes in the electrode absorption. The baseline shift as a function of potential for the ITO/ALO and ITO/ALO with 12 nm Au is shown in Figure S6. Before any spectra were measured, the electrode was cycled 10 times from −300 to 500 mV. (b) MCB spectra as a function of potential. The line shape is recreated by first measuring the difference spectra with respect to 0 mV and then holding the potential at −750 mV to desorb the monolayer and subtracting the resulting bleach from the difference spectra. (c, d) Changes in the peak frequency and absorbance vs the applied potential for the loss (negative peak) and gain (positive peak) features of the spectra.

For these reasons, the FTIR difference spectra in Figure 7a are shown in reference to the −400 mV spectra, approximately at *E*_pzc_. We can also indirectly measure the MCB spectrum as a function of potential by holding the potential at −750 mV for 15–20 min to desorb the monolayer after cycling the potential. The subsequent bleach feature from the loss of the monolayer can then be subtracted from the difference spectra to recreate the MCB spectrum. Here we see that there are subtle changes to the FTIR around *E*_pzc_ and interpret that as sensitivity to the surface charge. The overall blue-shift seen in the FTIR difference spectra when scanning the potential to positive values can be interpreted as an electrochromic shift induced in the carbonyl groups oriented perpendicular to the surface, with the more positive potentials giving rise to an electric field oriented toward the surface and contracting the equilibrium bond length, thereby increasing the frequency. There is also the possibility of other effects contributing to the response of the probe such as the direct polarization effect due to changes in the electron density of the molecule. This effect has been discussed in the case of 4-mercaptobenzonitrile monolayers, where the vibrational probe is directly conjugated to the surface through an aromatic group and is unlikely to be a major effect for the alkyl ketone studied here.^65,66^ For the present discussion, in the difference spectra, the decrease in absorption is termed the negative peak, while the increase in absorption is the positive peak. As illustrated in the plot in Figure 7c,d, the negative peak responds to the change in potential with a large shift in maximum frequency and a small change in intensity as shown with the red triangle markers. The positive peak responds in the opposite manner with a small shift in the maximum frequency but a large change in the intensity, and this is illustrated with the blue triangle markers.

**Figure 8:**
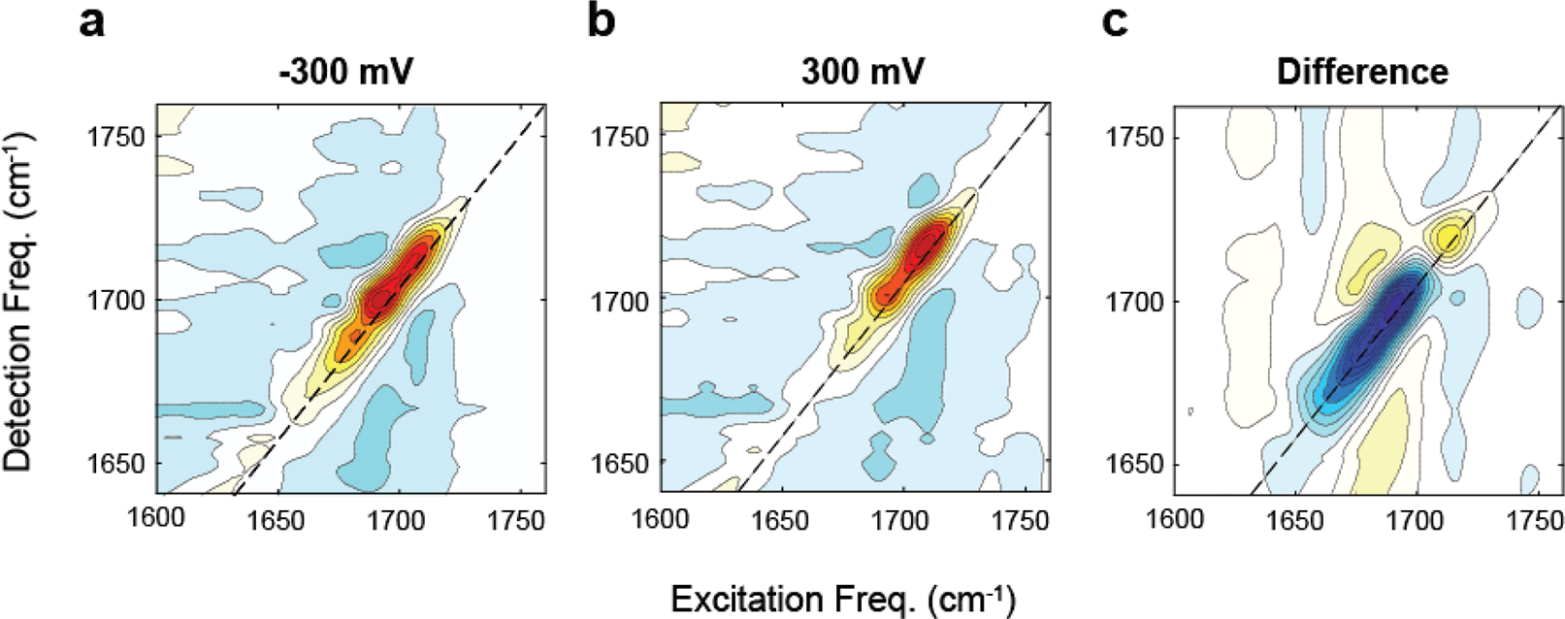
2D IR spectra of MCB on 10 nm Au layered on 1.2 nm Al_2_O_3_ and 20 nm ITO on Si ATR wafers are shown in (a) and (b) at −300 and 300 mV vs Ag/AgCl reference electrode, respectively, with 100 mM KCl supporting electrolyte in D_2_O. The difference spectrum of −300 mV subtracted off 300 mV is shown in (c).

Finally, we demonstrate the impact of the electrochemical potential on the 2D IR spectrum of MCB carbonyl. The 2D IR spectra at −300 and 300 mV vs Ag/AgCl are shown in Figure 8a,b, and the difference spectrum is shown in Figure 8c. The 2D spectra are collected on the same sample without removing or otherwise changing any parameters besides cycling the potential, and no adjustments to the signal intensities prior to taking the difference are necessary. Because of the longer acquisition time when measuring 2D IR spectra as compared to FTIR, we chose to collect the 2D spectra at potentials no greater than −300 or 300 mV vs Ag/AgCl to avoid degradation of the surface that may occur at longer time scales under an applied voltage. In Figure 8a,b, there is a clear change in the spectra that corresponds to the center frequency of the peak shifting. The change from more negative to positive potentials can be emphasized by taking the difference spectrum (Figure 8c) of the two spectra measured at −300 and 300 mV. Much of the baseline variation of the 2D spectrum is removed with subtraction, indicating that these features (which have also been observed in other potential-dependent 2D IR studies^67^) arise from static features not responsive to the applied voltage. In the difference spectrum, it is clear that the magnitude of the response is distinctly different from that shown in the FTIR, where the absorbance on the blue side was greatly increased when going from negative to positive potentials. In the 2D IR spectra, there is a large decrease in the red side of the difference spectrum but only a small increase on the blue side. These differences demonstrate that the FTIR and 2D IR spectroelectrochemical measurements highlight different aspects of the changes in the spectrum in response to the potential. However, a detailed discussion of the origin of these differences is beyond the scope of this study.

## Conclusions

In this study, we have discussed the general considerations and approach for designing and fabricating electrodes optimized for 2D IR spectroelectrochemical measurements with an emphasis on utilizing commercially available substrates and electrochemical cells to increase the accessibility of surface-specific ultrafast IR experiments. We have developed a layered electrode for performing 2D IR spectroelectrochemistry at the electrode–electrolyte interface that uses commercial Si ATR wafers with 20 nm of ITO and 1–2 nm Al_2_O_3_ grown via ALD, topped with 12 nm of sputtered Au. The ITO serves as the conductive layer for the working electrode, the Al_2_O_3_ as the adhesion layer between the ITO and Au, and the Au provides plasmonic enhancement that can readily be functionalized with thiols.

For the ketone–thiol studied in this work, MCB, we were able to measure the carbonyl response as a function of applied potential and observed trends similar to those of previous works in the linear and 2D vibrational response to interfacial electric fields with carbonyl stretches. While the vibrational response appears to shift in frequency linearly with applied field,^14,32−35^ we have also noted that the interfacial structure of the plasmonic film plays a large role in determining the line shape and degree of phase twist of the vibrational spectra, which leads to complications in interpreting the spectra of surface-bound and coupled vibrational reporters. This structure is dependent on both the average thickness of the plasmonic layer and the underlying substrate. We have also noted sensitivity to changes that occur around *E*_pzc_ which could provide another experimental tool for understanding how the EDL structures in response to the electrode surface charge.

There are other approaches to studying electrochemical interfaces with 2D IR, namely, different experimental geometries and substrate/electrode material choices, which come with intrinsic advantages and disadvantages. A major challenge of doing 2D IR in ATR mode is having to pass through a large amount of prism material. For common ATR materials, this becomes a problem because many of the high index of refraction materials are also highly dispersive or have small bandgaps that can lead to large background signals from free carriers that are excited when focusing IR beams into the material. Our approach uses thin Si wafers to minimize dispersion. We observe that it suffers from the problem of a large free carrier background only at higher pump powers and is not present in the spectra taken in this paper. CaF_2_ has been used as an ATR element^34^ to avoid the free carrier problem, but the index of refraction limits the use of the material for systems without concentrated electrolyte solutions, which approach the refractive index of CaF_2_.^68^

Other groups have avoided ATR mode and instead worked in the transmission geometry or in external reflectance.^32,69^ With these approaches, CaF_2_ can be used without concern for the refractive index of the substrate but with the technical challenge of keeping the path length through the cell sufficiently thin to reduce the solvent background while still thick enough to fit all the components of the electrochemical cell. All of the approaches except for the external reflection also use ITO as a conductive layer in addition to a plasmonic layer. A main reason for using ITO as the conductive layer is keeping the plasmonic layer sufficiently thin that the nanostructure with many small highly plasmonically active gaps is conserved. With external reflection, the absorption of the electrode and plasmonic materials is less of a concern, and the electrode material acts as a reflective mirror for the IR. This simplifies the experiment by allowing gold to be both the electrode and enhancement layer and removing the need for an adhesion layer. However, because the IR reflects externally, it passes through the solvent twice, doubling the absorption relative to a transmission cell of the same path length, exacerbating the background signal issue.

Overall, we believe that the ATR mode with layered electrodes provides the most flexibility for a variety of future experiments. While we expect that the electrode material used in our samples can be readily changed to study specific systems of interest, there is the unaddressed issue of the limits of using Au as the plasmonic material and the functionalizable material. This limits any tethered vibrational probes to ones that can coordinate with Au, such as thiols, which have low stability at longer chain lengths due to polymerization when they also contain functional groups that can act as reporters such as amines, carbonyls, and nitriles. Other functional groups that can coordinate with Au are citrates, which contain carbonyls that obscure the desired response from carbonyls positioned within the double layer, or amines, which coordinate much more weakly than either thiols or citrates.^70^ A possible solution to this constraint is the additional ALD layer such as Al_2_O_3_ on top of the Au that can be functionalized by other functional groups, such as silanes, and is sufficiently thin to allow for the plasmonic enhancement. The extensive library of commercially available silanes would open up a wider range of possible experiments, such as using varying chain lengths to extend the probe throughout the electric double layer to investigate how the rapidly changing electric field affects the hydrogen bonding and ion solvation structure and dynamics.^35^

The range of surface reactions that can be employed to build the required layers for electrochemical studies is very broad, and we hope that the general approach outlined in this paper can be utilized to study the wide range of interfacial electrochemical systems that have broad applicability, such as in areas of energy storage and heterogeneous catalysis, where molecular catalysts can be readily used or modified to serve as interfacial vibrational probes. At electrochemical interfaces, the reorganization of the solvent in the electric double layer plays a large role in accommodating charge transfer processes that occur at the interface.^71^ The ability of the solvent to reorient is strongly dependent on the strength of the interfacial electric field. We hope to directly probe the water structure and dynamics with electrochemical 2D IR to contribute to the understanding of how the water and ions in the EDL impact the electrochemical performance.

## Data Availability

All data has been made publically available on Zenodo at https://zenodo.org/doi/10.5281/zenodo.10210850.

## Supporting Information Available

Additional data processing information, AFM images of samples, and control experiments (PDF)
