# Supplementary material for: Development and Characterization of Electrodes for Surface-Specific Attenuated Total Reflection Two-Dimensional Infrared Spectroelectrochemistry
Source: J Phys Chem C Nanomater Interfaces. 2023 Nov 28;127(48):23199–211. doi: 10.1021/acs.jpcc.3c05445 (PMC10711796; doi:10.1021/acs.jpcc.3c05445)
Supplement: Supplementary file 1 — jp3c05445_si_001.pdf [file jp3c05445_si_001.pdf]

Supplementary Information for Development and Characterization of Electrodes for Surface-Specific Attenuated Total Reflection Two-Dimensional Infrared Spectroelectrochemistry

Melissa Bodine,[†] Vepa Rozyyev,[‡] Jeffrey W. Elam,[¶] Andrei Tokmakoff,[†] and Nicholas H. C. Lewis[*,†]

†*Department of Chemistry, James Franck Institute, and Institute for Biophysical Dynamics, The University of Chicago, Chicago, IL, 60637, USA.*

‡*Pritzker School of Molecular Engineering, The University of Chicago, Chicago, IL, 60637, USA.*

¶*Applied Materials Division, Argonne National Laboratory, Lemont, IL, 60439, USA.*

E-mail: nlewis@uchicago.edu

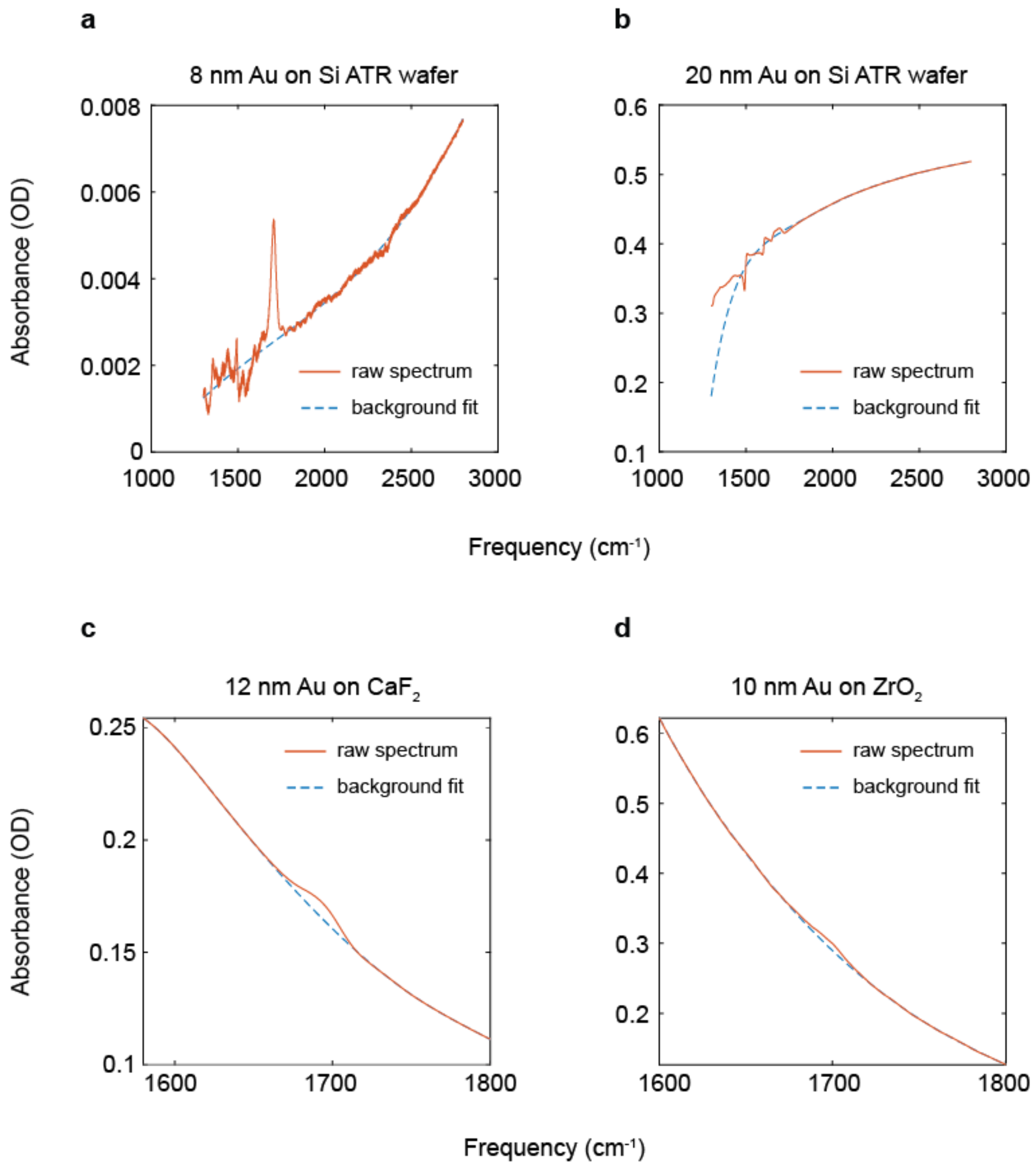

Figure S1: Raw FTIR spectra and the baseline fitting for MCB functionalized Au films on different substrates. For Si ATR wafer substrates the spectra for 4 and 8 nm Au films were fit with a third order polynomial from 1800 to 2800 cm^{-1} and extrapolated down to 1300 cm^{-1} for background subtraction. (a) An example of the background fitting shown for 8 nm of Au on Si ATR wafers. For thicker films (12 nm and above), the spectra taken with the Si ATR wafers was fit with a seventh order polynomial from 1450 to 2800 cm^{-1}, excluding the region from 1451 to 1850 cm^{-1}. (b) Example of background fitting for 20 nm of Au on Si ATR wafers. For the CaF_2 and ZrO_2 substrates, the background for all spectra regardless of Au film thickness was fit with a sixth order polynomial from 1580 to 1800 cm^{-1} excluding the region from 1670 to 1730 cm^{-1}. (c-d) Example background fitting for 12 nm Au on CaF_2 and 10 nm Au on ZrO_2.

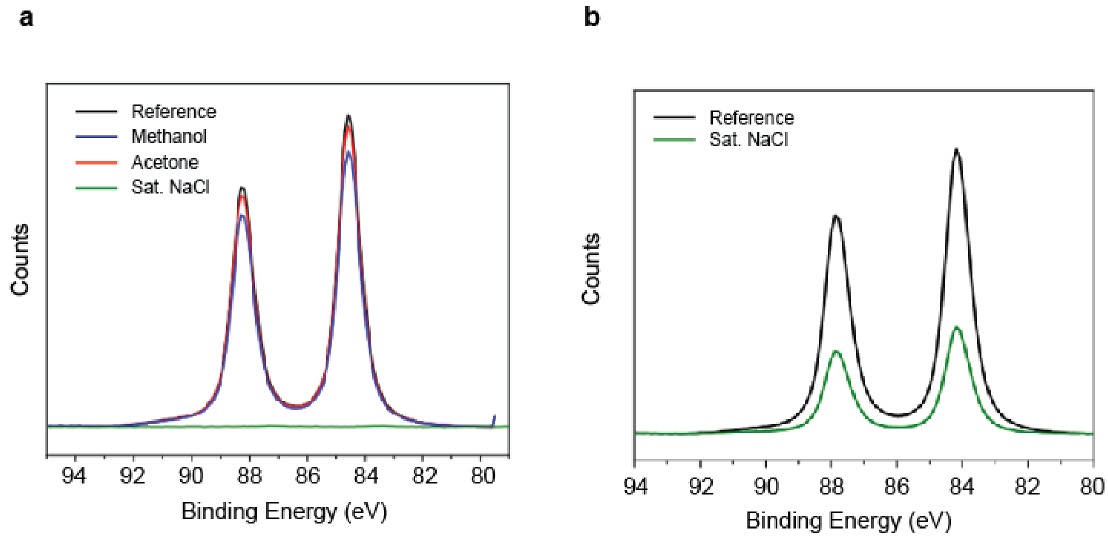

Figure S2: XPS peaks of Au4f showing the change in peaks for (a) Au on 20nm of ITO and (b) Au on 1.2 nm of ALO on ITO.

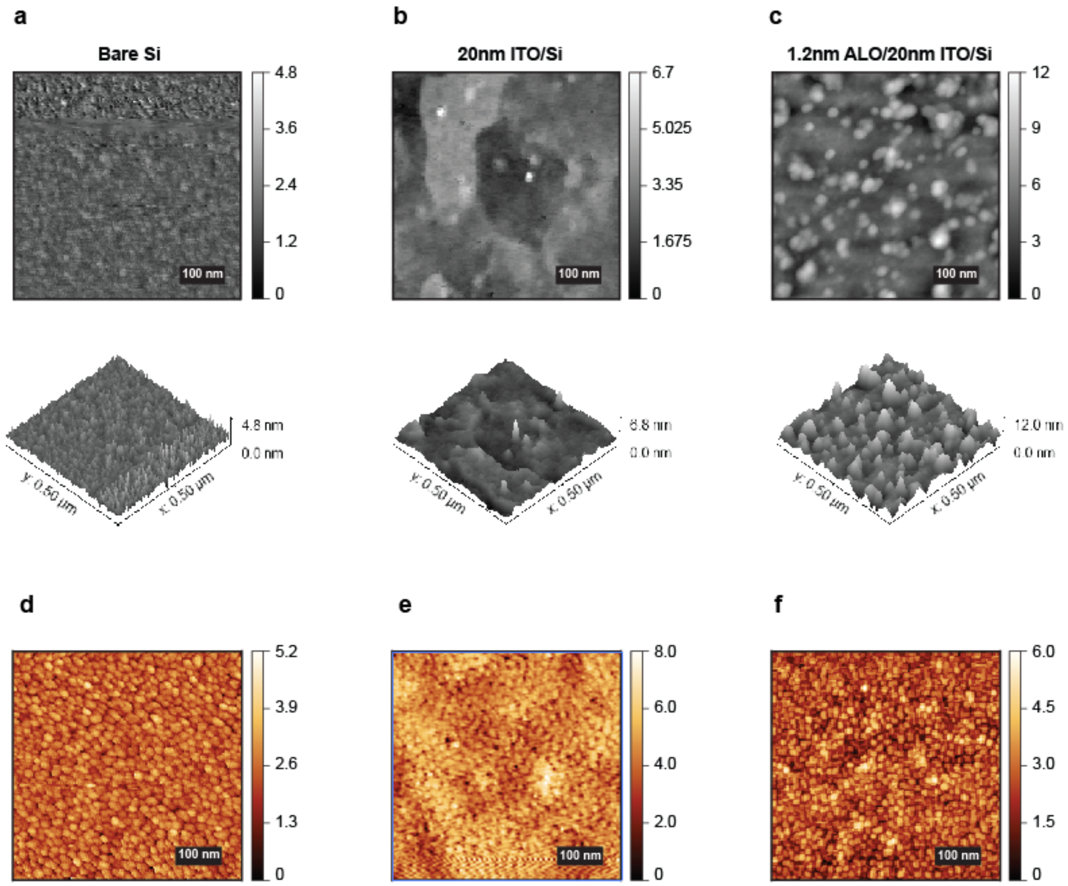

Figure S3: AFM images of (a) bare Si wafers, (b) 20 nm ITO grown via ALD on Si, and (c) 1.2 nm of ALO grown via ALD on 20 nm of ITO on Si. (d-f) AFM images of the same substrates with 8 nm of Au sputtered on top. The structure of Au is noticeably different on ITO than the other substrates, and forms an interconnected pattern rather than separate islands.

a

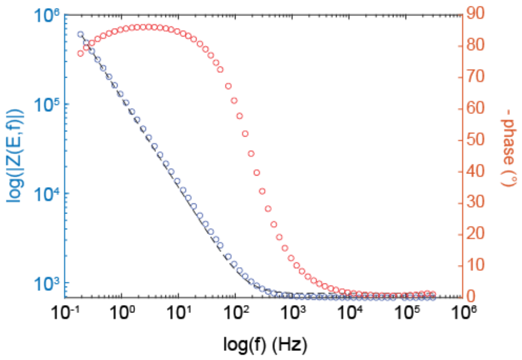

Figure S4: (a) Example plot of the modulus of impedance, $|Z(E, f)|$, shown on the left y axis as a function of the AC frequency, f, in Hz and corresponding fit shown with the black dashed line. The negative phase is plotted on the right y axis.

a

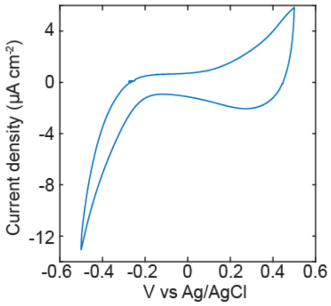

Figure S5: (a) Cyclic voltammogram of the layered electrode (12 nm Au/1.2 nm Al_2O_3/20 nm ITO on Si ATR wafers) functionalized with MCB. Supporting electrolyte is 100 mM $KClO_4$ in H_2O.

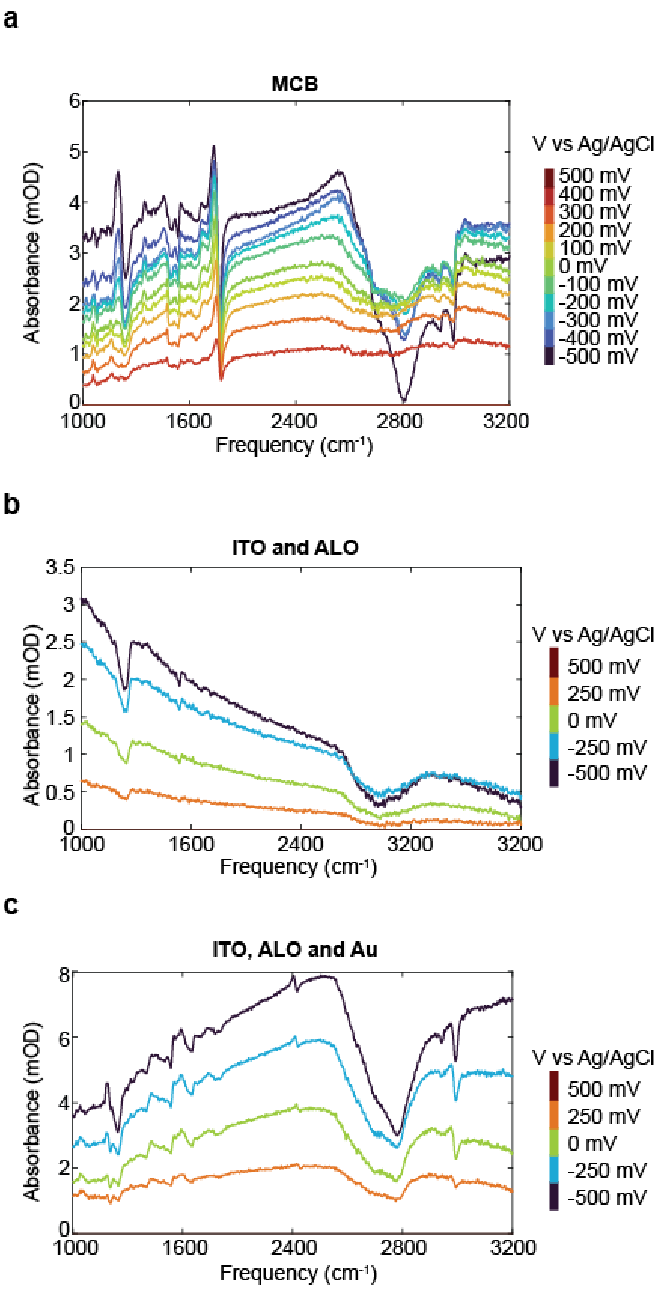

Figure S6: (a-c) Raw difference spectra shown in reference to the 500 mV spectrum to show the increasing absorbance with more negative potentials. Supporting electrolyte is 100 mM KCl in D_2O. The baseline shift that is in the spectra for the (a) MCB functionalized electrode is shown to also be present in both (b) ITO/ALO samples and (c) ITO/ALO with 12nm of Au. Because it is a material response it is subtracted from the difference spectra shown in the main text for clarity.
